# The longitudinal psychological pathway from family support to entrepreneurial intention: a mediation model of entrepreneurial self-efficacy among art and design students

**DOI:** 10.3389/fpsyg.2026.1745833

**Published:** 2026-03-19

**Authors:** Li Yue, Pang Shengnan, Zhao Lu, Hashem Salarzadeh Jenatabadi

**Affiliations:** 1 School of Art and Design, Shandong Women’s University, Jinan, Shandong, China; 2 Huace Film Academy, Communication University of Zhejiang, Tongxiang, Zhejiang, China; 3 Department of Digital Media, Shenzhen Polytechnic University, Shenzhen, Guangdong, China; 4 School of Business, Monash University Malaysia, Subang Jaya, Selangor, Malaysia; 5 Department of Science and Technology Studies, Faculty of Science, University Malaya, Kuala Lumpur, Malaysia

**Keywords:** entrepreneurial behavior, entrepreneurial self-efficacy, extended theory of planned behavior, family support, social cognitive theory

## Abstract

**Introduction:**

Entrepreneurial intention is increasingly recognized as an important outcome within creative disciplines, yet limited longitudinal evidence exists regarding the social and psychological factors associated with its development among art and design students.

**Methods:**

Drawing on the extended Theory of Planned Behavior and Social Cognitive Theory, this study examines the longitudinal associations between family support, entrepreneurial self-efficacy, perceived behavioral control, and entrepreneurial intention among Chinese art and design students. Using a three-wave survey design, data were collected from 786 students enrolled in art and design programs across three provinces in China. Structural equation modeling was employed to examine direct and indirect associations among the study variables over time.

**Results:**

The results indicate that entrepreneurial attitude and PBC are positively associated with entrepreneurial intention, whereas subjective norm shows no significant association. Family support is positively associated with entrepreneurial intention and is also related to higher levels of entrepreneurial self-efficacy and PBC. Moreover, entrepreneurial self-efficacy partially mediates the longitudinal association between family support and entrepreneurial intention, highlighting its role as a key psychological mechanism linking social support with entrepreneurial motivation.

**Discussion:**

By integrating extended Theory of Planned Behavior and Social Cognitive Theory within a longitudinal framework, this study advances understanding of how social context and self-beliefs jointly relate to entrepreneurial intention in creative education. The findings underscore the relevance of family support and entrepreneurial self-efficacy in art and design contexts and offer implications for entrepreneurship education and support strategies tailored to creative disciplines.

## 1 Introduction

Entrepreneurial studies are important because they are associated with economic development through innovation, job creation, and economic growth (Gomes et al., 2025). A strong entrepreneurial environment is often linked to the emergence of new sectors and the revitalization of existing ones. In this context, China has emerged as a global leader, with a strong emphasis on entrepreneurial activities (Cai et al., 2025). The Chinese government’s policies and programs are intended to promote entrepreneurship, recognizing its potential to drive economic growth. China hopes to maintain its competitive edge in the global market and assure long-term economic growth by encouraging entrepreneurship.

Universities play an important role in the entrepreneurial ecosystem, acting as incubators for innovative ideas and future company leaders. However, it is critical to note that different disciplines of study necessitate distinct methods to entrepreneurial education. The diversity of academic specialties makes a one-size-fits-all strategy to cultivating entrepreneurial impulses inefficient (Bort et al., 2024). Each area, whether engineering, business, or the arts, has distinct qualities and demands that shape how entrepreneurship should be taught and promoted. Art and design students, for example, follow a distinct school of thought that emphasizes originality, innovation, and aesthetic value. Nabi et al. (2017) found that entrepreneurial attitudes and intents differ significantly across fields, highlighting the necessity for specialized training approaches. These students approach issues and opportunities differently than those in more traditional business disciplines, demanding a specialized educational approach that values and leverages their distinct perspectives.

Understanding the key factors associated with entrepreneurial intention among art and design students is important for informing policy development and institutional support strategies. By examining these students’ individual needs and motivations, policymakers and educational institutions can develop focused programs to encourage their entrepreneurial spirit. Anggadwita and Dhewanto (2016) found that perceived behavioral control (PBC) and family support are significantly related to entrepreneurial intention, illustrating the multidimensional nature of entrepreneurial decision-making. This study seeks to provide a thorough understanding of these aspects, as well as insights that will aid in developing successful programs to advise and support future entrepreneurs in the arts and design sectors. Such tailored support is critical for realizing these students’ full potential, ensuring that their creative talents lead to successful entrepreneurial initiatives that contribute to economic growth and innovation.

## 2 Literature review

### 2.1 Entrepreneurial intention in art and design students

The rapid expansion of the creative economy has heightened the strategic importance of entrepreneurship in art and design. Advances in digital technologies have broadened the scope of creative work beyond traditional tangible outputs to include service design, user experience design, and the integration of digital and physical products (Zeng et al., 2023). These developments have been associated with new entrepreneurial opportunities for creative professionals, while simultaneously introducing heightened uncertainty and competition within creative labor markets. In this context, entrepreneurial pathways have become an increasingly salient career option for art and design students (Otchengco and Akiate, 2021).

Prior research suggests that art and design education equips students with transferable creative and technical skills that can be applied to entrepreneurial activities across multiple creative industries, including graphic design, fashion, product development, and media production (Elfeky and Elbyaly, 2021). However, rather than uniformly translating into entrepreneurial intention, these skills interact with contextual and psychological factors that shape students’ willingness to pursue entrepreneurial careers. Compared with students in business or engineering disciplines, art and design students often face less standardized career trajectories, greater income uncertainty, and stronger dependence on project-based or freelance work, which may influence how entrepreneurial intention are formed and sustained.

Despite growing interest in creative entrepreneurship, empirical research on entrepreneurial intention among art and design students remains fragmented and limited in scope. Existing studies often generalize findings from business-oriented samples or focus on skill development without adequately examining motivational and psychological mechanisms specific to creative disciplines (Yu and Jiang, 2021). Moreover, findings on the factors associated with entrepreneurial intention in art and design education are inconsistent, particularly regarding the roles of social support and self-belief. This gap is especially evident in higher vocational and practice-oriented art and design programs, where students may possess strong creative capabilities but face structural and psychological barriers to entrepreneurship.

Taken together, the literature indicates a need for more focused investigation into how entrepreneurial intention develops among art and design students, with particular attention to discipline-specific contexts and psychological processes. Addressing this gap can contribute to a more nuanced understanding of creative entrepreneurship and inform the design of educational and support interventions that align with the unique characteristics of art and design education.

### 2.2 Family support and entrepreneurship

Family support has been widely recognized as a key social context associated with entrepreneurial intention, particularly in collectivist societies such as China, where family relationships strongly influence career decision-making and risk-related choices (Khayru et al., 2021). In this cultural setting, family support extends beyond emotional encouragement to include financial assistance, practical guidance, and implicit risk-sharing, all of which are linked to lower perceived uncertainty in entrepreneurial pursuits. Rather than functioning as a uniform resource, family support operates through multiple channels that jointly relate to individuals’ perceptions of entrepreneurship as a viable and acceptable career option.

Empirical studies consistently report a positive association between family support and entrepreneurial intention among university students, although the underlying mechanisms vary across contexts. Prior research suggests that emotional reassurance from family members is associated with higher confidence and lower fear of failure, while financial and instrumental support may be related to stronger perceptions of feasibility during early stages of venture consideration (Kong et al., 2020; Li et al., 2023; Lyu et al., 2024). More recent work further indicates that family support may be indirectly associated with entrepreneurial intention through psychological resources such as self-efficacy, thereby influencing how individuals evaluate their own entrepreneurial capabilities (Chen, 2024; Zhou et al., 2024).

Despite broad agreement on the relevance of family support, existing studies often treat it as a direct antecedent of entrepreneurial intention, with limited attention to its interaction with individual psychological processes. Moreover, much of the literature focuses on general student populations, leaving open questions regarding how family support functions in disciplines characterized by higher uncertainty and less conventional career pathways (Tran et al., 2024). In creative fields such as art and design, where entrepreneurial outcomes are often unpredictable and socially contested, family support may be particularly salient in relation to entrepreneurial aspirations and perceived risks. This suggests the need for research that moves beyond descriptive associations to examine how family support is psychologically internalized and translated into entrepreneurial intention within discipline-specific contexts.

### 2.3 Self-efficacy role in entrepreneurial intention studies

Self-efficacy, defined as individuals’ beliefs in their capability to perform tasks and manage challenges, has been widely recognized as a central psychological resource in entrepreneurship (Bandura, 1977). In entrepreneurial contexts, self-efficacy is related to how individuals evaluate opportunities, respond to uncertainty, and persist in the face of obstacles. Prior research consistently shows that individuals with stronger entrepreneurial self-efficacy are more likely to report higher entrepreneurial intention, reflecting greater confidence in their perceived ability to initiate and sustain entrepreneurial activity (Al-Qadasi et al., 2023; Ferreira-Neto et al., 2023).

Rather than operating in isolation, self-efficacy is associated with entrepreneurial intention through its links with motivational and behavioral orientations. Empirical evidence suggests that higher self-efficacy is associated with greater tolerance for risk, stronger commitment to entrepreneurial goals, and increased willingness to invest effort and resources in venture development (Christensen et al., 2023; Ciptono et al., 2023; Wardana et al., 2024). These beliefs are related to proactive problem-solving and adaptive responses to uncertainty, which are particularly salient in early-stage entrepreneurial decision-making (Oulhou and Ibourk, 2023).

At the same time, the literature emphasizes that self-efficacy functions within a broader social and contextual environment. Access to resources, social support, educational experiences, and market conditions can shape the extent to which efficacy beliefs are associated with entrepreneurial intention (Taneja et al., 2024). Personal attributes such as resilience and creativity further interact with self-efficacy, shaping how individuals interpret entrepreneurial challenges and opportunities (Gregori et al., 2024; Pham and Le, 2023). Accordingly, entrepreneurial intention is best understood as the outcome of dynamic interactions between self-efficacy and contextual factors, rather than as a product of individual confidence alone. This perspective underscores the importance of examining how psychological beliefs are shaped by social and environmental conditions in entrepreneurship research.

### 2.4 Theoretical framework

#### 2.4.1 Theory of planned behavior

The Theory of Planned Behavior (TPB) is a key framework in entrepreneurial intention research, offering insight into psychological factors associated with entrepreneurial intentions. According to TPB, entrepreneurial intention is shaped by attitudes toward entrepreneurship, subjective norms, and PBC (Ajzen, 1991) [21]. Existing research provides support for these components. For example, Munir et al. (2019) reported that positive views toward entrepreneurship and higher levels of PBC are positively associated with entrepreneurial intention among university students. Similarly, Al-Qadasi et al. (2021) found that subjective norms, including family and peer support, are significantly related to entrepreneurial intention in the Yemeni context. In addition, Otchengco and Akiate (2021) reported that PBC is strongly associated with entrepreneurial intention, highlighting the relevance of self-efficacy and perceived resource availability. Collectively, these studies support the applicability of TPB across cultural contexts and illustrate its usefulness for understanding psychological factors related to entrepreneurial intention. TPB further provides a conceptual basis for informing educational programs and support systems aimed at fostering entrepreneurial intention by addressing attitudes, social influences, and perceived control.

#### 2.4.2 Social cognitive theory

Albert Bandura’s Social Cognitive Theory (SCT) has been widely applied in entrepreneurial intention research, emphasizing the interaction of personal, behavioral, and environmental factors in relation to entrepreneurial beliefs and intentions. Within SCT, self-efficacy, defined as belief in one’s capability to perform specific tasks, is considered a central psychological construct associated with entrepreneurial intention. Empirical studies provide support for this perspective. For instance, Liu et al. (2022) found that self-efficacy is positively related to entrepreneurial intention among Chinese university students. Similarly, Munir et al. (2019) reported that higher self-efficacy is associated with stronger entrepreneurial intention across both emerging and developing economies. In addition, Al-Qadasi et al. (2021) observed that self-efficacy, alongside attitudes and PBC, is significantly related to entrepreneurial intention among Yemeni youth. Together, these findings support the relevance of SCT as a theoretical framework for understanding entrepreneurial intention by highlighting the role of self-efficacy and its interaction with contextual factors (Otchengco and Akiate, 2021; Vamvaka et al., 2020).

### 2.5 Entrepreneurial intention in art and design students

The art and design sector is witnessing a growing emphasis on innovation and entrepreneurship among professionals, which is widely discussed as being relevant to the future development of the design industry and the expansion of creative activities. The rapid development of the digital industry has been associated with substantial transformations in industrial structures. The scope of creative design has broadened to encompass not only conventional tangible products, but also the integration of software and hardware, service design, and user experience design (Zeng et al., 2023). These developments have introduced new opportunities as well as notable challenges for innovation and entrepreneurial engagement among design professionals. Otchengco and Akiate (2021) observed that the creative economy has emerged as a significant catalyst for global transformation. This trend presents growth opportunities for students pursuing art and design majors and has encouraged higher vocational institutions to place greater emphasis on cultivating practical skills relevant to innovation and entrepreneurship (Passavanti et al., 2023).

Art and design programs provide students with essential skills across various domains, including graphic design, illustration, fashion design, and related fields (Zeng et al., 2023). These skills may be applied to entrepreneurial activities. For example, in graphic design, students learn to create visual content for print and digital media, which may support the establishment of graphic design studios or platforms offering branding services and custom design templates (Guerra-Tamez, 2023). Illustration programs develop competencies in sketching, digital art, and visual storytelling, which may be applied in freelance illustration work or the creation of customized products such as illustrated garments. Fashion design education includes garment construction, textile selection, and trend analysis, which are relevant to entrepreneurial activities such as launching a clothing line or operating a boutique (Elfeky and Elbyaly, 2021). Product design skills support the creation of functional and esthetically appealing objects and are associated with opportunities in areas such as home goods and eco-friendly packaging solutions (Cao, 2024). Interior design programs focus on developing functional and visually appealing interior environments, which may be extended into professional services such as design consultation or custom home décor offerings (Afacan, 2016).

Art and design students are also frequently exposed to networking opportunities involving peers, faculty members, and industry professionals. The development of professional networks is often regarded as important in the early stages of creative enterprise development. Many graduates pursue careers in creative industries, including creative product development, advertising design, art photography, media, film, and television (Zhao et al., 2022). Despite this, research specifically examining entrepreneurial intention among students in higher vocational art and design programs remains limited. Existing studies report inconsistent findings, and there is a lack of consensus regarding factors associated with entrepreneurial intention in this population. The distinctive characteristics of art and design students in higher vocational settings may present particular challenges related to the formation and sustainability of entrepreneurial intention, underscoring the need for further investigation in this area (Yu and Jiang, 2021).

Accordingly, research that examines the current state of entrepreneurial intention among higher vocational art and design students and explores contextually appropriate support strategies may contribute to improved understanding of entrepreneurial education in creative fields. Such efforts may help identify ways to support students’ development of entrepreneurial competencies and readiness to engage in entrepreneurial activities.

### 2.6 Gaps of previous studies

Existing research on entrepreneurial intention has largely focused on students in business and economics disciplines, often overlooking the distinctive contexts and career pathways of art and design students. This imbalance is noteworthy given that creative disciplines are characterized by greater uncertainty, non-linear career trajectories, and stronger reliance on personal and social resources. As a result, findings derived from traditional business-oriented samples may not fully capture the factors associated with entrepreneurial intention in creative education contexts.

Moreover, much of the existing literature relies on cross-sectional research designs, which limit insight into how entrepreneurial intention and its psychological antecedents develop over time. Although studies have highlighted the importance of psychological factors such as entrepreneurial self-efficacy and motivation, few have examined how these constructs evolve and interact across different stages of students’ educational trajectories. Longitudinal evidence remains scarce regarding how family support is internalized over time and how it is associated with changes in entrepreneurial self-efficacy and entrepreneurial intention.

In addition, research integrating psychological perspectives with social and contextual influences remains underdeveloped in studies of creative entrepreneurship. While family support is widely recognized as a salient factor in collectivist cultures such as China, limited attention has been given to its dynamic relationship with entrepreneurial self-efficacy in relation to entrepreneurial intention among art and design students. Addressing these gaps through longitudinal and interdisciplinary approaches can provide a more nuanced understanding of the temporal and psychological processes linked to entrepreneurial intention and inform more targeted educational and policy interventions in creative fields.

### 2.7 Current study

The present study examines the longitudinal associations between family support, entrepreneurial self-efficacy, and entrepreneurial intention among art and design students. Given the distinctive creative orientation and non-linear career pathways characteristic of this population, the study examines how family support functions as a salient social context over time and how it is associated with changes in students’ entrepreneurial self-beliefs and intentions. By adopting a multi-wave design, the study moves beyond static associations and provides insight into the temporal dynamics characterizing the associations between family support, psychological resources, and entrepreneurial intention in creative disciplines.

To establish a coherent theoretical foundation, this study integrates the extended TPB and SCT. The extended TPB incorporates key social and cognitive factors, including family support, PBC, and subjective norms, while SCT highlights entrepreneurial self-efficacy as a central psychological resource. Integrating these perspectives allows for a more comprehensive examination of how external social support and internal efficacy beliefs jointly relate to entrepreneurial intention over time.

The central focus of the study is the mediating role of entrepreneurial self-efficacy in the longitudinal association between family support and entrepreneurial intention. Rather than treating family support solely as a direct antecedent, the study examines how perceived family support is associated with subsequent self-efficacy levels, which in turn relate to later entrepreneurial intention. By emphasizing this temporal mediation process, the study aims to clarify the psychological pathways linking social support with entrepreneurial motivation among art and design students. This approach contributes to a more nuanced understanding of entrepreneurial intention development and offers implications for designing educational and support interventions that strengthen self-efficacy within creative education contexts.

## 3 Research hypotheses development

### 3.1 Entrepreneurial attitude

The initial determinant that is most relevant to an individual’s entrepreneurial intention within the TPB framework is attitude. According to Ajzen (1991), attitude is conceptualized as a psychological inclination that may manifest as either a positive or negative orientation and involves the evaluation of a given behavior. Ajzen (2001) further defined entrepreneurial attitude as the extent to which an individual holds a favorable or unfavorable personal evaluation of entrepreneurship. Esfandiar et al. (2019) noted that attitudes toward entrepreneurship reflect individuals’ beliefs and their perceptions of the potential positive or negative outcomes associated with entrepreneurial activity. Cui and Bell (2022) similarly suggested that a favorable attitude toward entrepreneurship is associated with greater engagement in entrepreneurial pursuits. Based on this literature, the first hypothesis is proposed as follows:

*H1*: Entrepreneurial attitude is positively associated with entrepreneurial intention.

### 3.2 Subjective norm

The second indicator of the TPB is subjective norm, which pertains to the perceived social pressure that individuals may experience when engaging in a particular behavior. Subjective norm refers to the perceived influence of individuals’ social environment, including friends, instructors, spouses, or parents, on their behavioral intentions. Ajzen (1991) proposed that subjective norm reflects the collective set of normative beliefs held by these significant social referents. Previous research on entrepreneurial intention within the TPB framework has highlighted the relevance of subjective norms within conceptual models. Notably, scholars such as Yasir et al. (2022), He and Ding (2023), and Ho et al. (2022) have reported that subjective norm demonstrates a limited explanatory association with entrepreneurial intention. Drawing upon this existing body of research, the following hypothesis is proposed.

*H2*: Subjective norm is positively associated with entrepreneurial intention.

### 3.3 PBC

Perceived behavioral control (PBC) is a fundamental element within the TPB framework and is commonly applied in the examination of entrepreneurial intention. PBC refers to an individual’s subjective assessment of their capability to perform a particular behavior or to overcome barriers that may hinder engagement in that behavior, specifically in entrepreneurial contexts (Vamvaka et al., 2020). PBC has been widely examined in relation to entrepreneurial intention, as it reflects individuals’ perceptions of feasibility and control when considering entrepreneurial activities. The entrepreneurial context is shaped by multiple external factors, including economic conditions, market competition, and government policies and regulations (Aga, 2023). Individuals reporting higher levels of PBC often perceive themselves as better able to adapt to and manage environmental demands.

Perceived access to resources represents an important component of PBC. Access to financial resources, professional networks, and relevant information is frequently discussed in the entrepreneurship literature. When individuals perceive that such resources are available to them, this perception is often associated with higher confidence in entrepreneurial engagement. Accordingly, PBC is frequently regarded by researchers and educators as a key construct for understanding entrepreneurial intention (Aliedan et al., 2022; Juan et al., 2022; Sargani et al., 2021). Building on this literature, the present study examines the role of PBC within the Chinese context.

*H3*: Perceived behavioral control is positively associated with entrepreneurial intention.

### 3.4 Family support

For individuals considering entrepreneurial activities, psychological readiness to cope with obstacles and challenges is important. Greater family support is often associated with higher confidence and a greater willingness to confront difficulties encountered in entrepreneurial pursuits. According to Saoula et al. (2023), family support may serve as a source of inspiration and motivation for individuals’ goals. Individuals raised in business-oriented families may perceive their parents as role models in relation to entrepreneurial intention (Halizah and Mardikaningsih, 2022; Tran et al., 2024). Beyond immediate family, acquaintances and relatives are also frequently discussed as relevant social influences in entrepreneurial contexts (Lyu et al., 2024; Peris-Delcampo et al., 2023). Such social connections may provide informational, financial, advisory, or emotional support that is associated with the development and continuation of entrepreneurial ideas (Chen, 2024; Younis et al., 2021). As a result, family support has been widely examined in the literature as a key variable related to entrepreneurial intention (Chaudhuri et al., 2023; Molino et al., 2018).

Family support has also been examined in relation to entrepreneurial self-efficacy, defined as individuals’ confidence in their perceived ability to initiate and manage entrepreneurial activities. When family members provide encouragement and express confidence, this support is often associated with higher levels of perceived competence and self-belief. Prior research suggests that emotional support from family members, including empathy and understanding during challenging periods, is related to more positive self-perceptions and stronger self-efficacy beliefs (Suhartanto, 2022). A supportive family environment may be associated with more effective coping with stress and uncertainty in entrepreneurial contexts. In addition, family members with entrepreneurial experience may serve as sources of informational guidance, which can enhance individuals’ understanding of entrepreneurial processes and is associated with higher levels of self-efficacy through perceived preparedness and capability (Ferreira-Neto et al., 2023).

Family support has also been discussed in relation to PBC, a core construct within the TPB and the Theory of Reasoned Action. Forms of family support such as financial assistance or resource sharing may be associated with perceptions of feasibility and control over entrepreneurial activities. Such support may be linked to lower perceived barriers related to resource constraints. Powell and Eddleston (2017) noted that emotional support from family members is associated with greater confidence and lower perceived stress in entrepreneurial contexts. When individuals perceive emotional support from their family, they may be more likely to report greater control over entrepreneurial challenges, which is reflected in higher levels of PBC (Awal et al., 2023). Based on the preceding discussion, the following hypotheses are proposed:

*H4*: Family support is positively associated with entrepreneurial intention.

*H5*: Family support is positively associated with entrepreneurial self-efficacy.

*H6*: Family support is positively associated with PBC.

### 3.5 The mediating role of entrepreneurial self-efficacy

Barbaranelli et al. (2019) define self-efficacy as an individual’s confidence in their ability to perform goal-oriented tasks. Newman et al. (2019) associate self-efficacy with individuals’ tendencies to pursue personal objectives. Self-efficacy is a central construct derived from SCT. Bandura (1985) proposed that individual behavior is related to the interaction of personal, behavioral, and environmental factors. According to Al-Qadasi et al. (2023), the interrelations among these factors are associated with individuals’ confidence in managing specific behaviors and their expectations regarding behavioral outcomes.

Prior research has consistently identified self-efficacy as an important psychological construct associated with entrepreneurial intention and behavior (Christensen et al., 2023; Pukkinen et al., 2023). An expanding body of literature within entrepreneurial intention and behavioral models further suggests that self-efficacy frequently functions as an intervening variable linking contextual and individual factors in entrepreneurship and social psychology (Saoula et al., 2023). Murad et al. (2022) and Thein et al. (2023) reported that self-efficacy is closely associated with behavioral orientations through cognitive processes, goal-setting tendencies, and outcome expectations. Higher levels of entrepreneurial self-efficacy are often associated with perceiving entrepreneurship as a viable and attractive career option (Pham and Le, 2023; Pukkinen et al., 2023). When individuals report stronger confidence in their perceived ability to initiate and manage a business, they are more likely to express entrepreneurial intention rather than preference for traditional employment. In addition, Broccia et al. (2022) noted that entrepreneurs with higher task-specific self-efficacy are more likely to engage in entrepreneurial activities than those with lower self-efficacy.

As discussed in the preceding section, family support has been examined in relation to entrepreneurial self-efficacy. Accordingly, family support may be associated with entrepreneurial intention both directly and indirectly through its association with entrepreneurial self-efficacy. This mediation framework proposes that perceived family support is linked to entrepreneurial intention via differences in self-efficacy beliefs. Based on this conceptual reasoning, the following hypotheses are proposed:

*H7*: Entrepreneurial self-efficacy is positively associated with perceived behavioral control.

*H8*: Entrepreneurial self-efficacy is positively associated with entrepreneurial intention.

*H9*: Entrepreneurial self-efficacy mediates the association between family support and entrepreneurial intention.

**Figure 1:**
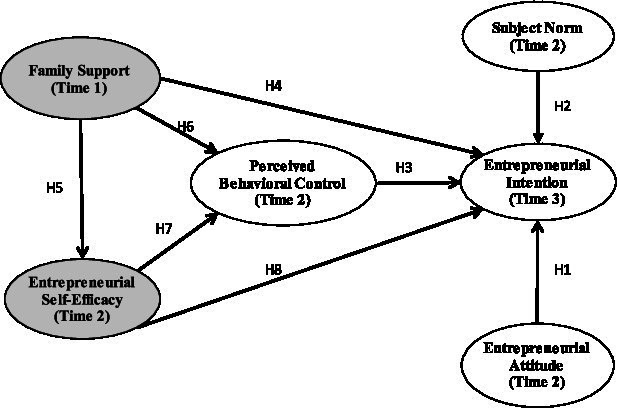
Research model.

Figure 1 shows our research framework.

## 4 Materials and methods

### 4.1 Measures

In this study, we measure the variables in the research model with pre-existing instruments because their validity and reliability have been demonstrated in numerous prior empirical studies. On a 7-point Likert scale, all questionnaire items are scored from “1 strongly disagree” to “7 strongly agree.”

There are two main approaches for assessing latent variables: (a) Dimension Scores and (b) Random Parceling. The dimension technique creates latent variables from observable data that are thought to represent underlying dimensions. This method is founded on the concept that specific observable indicators represent larger, unseen entities. Random parceling, on the other hand, is the process of randomly combining numerous observed variables to create indicators for latent variables. The argument for this strategy is that merging many things into parcels decreases measurement error and improves the latent construct’s dependability. In our study, we used dimension scores since earlier research had previously characterized each latent variable, providing a clear foundation for our analysis.

#### 4.1.1 Entrepreneurial intention

Entrepreneurial intention is the primary dependent variable in our study and effectively assessing it necessitates a complete grasp of its complexities and ramifications. Previous research has shown the significance of entrepreneurial intention as a predictor of entrepreneurial action, making it an important topic for both scholars and policymakers. The measurement of entrepreneurial intention has evolved throughout time, with numerous scales and approaches devised to capture this complicated concept. Liñán and Chen (2009) established a comprehensive scale to assess entrepreneurial intention, taking into account personal attitude, PBC, and subjective norms. This measure has been widely utilized and validated in a variety of cultural situations, providing a reliable framework for assessing entrepreneurial intent. Similarly, Anggadwita and Dhewanto (2016) added to the field by fine-tuning and customizing existing scales to better suit certain populations and circumstances, ensuring that the measures are both reliable and valid. We updated the entrepreneurial intention scale from Liñán and Chen (2009) and Anggadwita and Dhewanto (2016) to appropriately reflect our target population’s intentions. The modified scale comprises of five questions aimed at capturing the essence of entrepreneurial intention. This scale’s sample questions are “I am ready to do anything to be an entrepreneur,” “I will make every effort to start and run my own business,” and “I have a very serious thought of starting a business.” These questions are designed to assess the respondents’ preparation, dedication, and seriousness about entrepreneurship, offering a comprehensive measure of their entrepreneurial intention.

The factor loadings of all survey questions were between 0.723 and 0.839, suggesting that there were robust relationships between the questions and their underlying constructs. The scale’s internal consistency was evident in the construct reliability value of 0.858, which was significantly higher than the acceptable threshold of 0.60. Furthermore, the scale’s construct validity and its capacity to differentiate between various constructs were further substantiated by the average variance extracted (AVE) value of 0.567, which exceeded the minimum requirement of 0.5. The scale’s goodness of fit indices was also noteworthy, with a GFI of 0.934, AGFI of 0.988, NFI of 0.904, IFI of 0.927, CFI of 0.934, and RMSEA of 0.033. These values suggest that the scale is a reliable and robust instrument for measuring the intended constructs, as it has an excellent fit with the data.

#### 4.1.2 Entrepreneurial attitude

A comprehensive foundation for measuring entrepreneurial attitude has been established by prior research, which is essential for comprehending the predispositions of individuals toward entrepreneurship. Nabi et al. (2011) conducted a significant study in this field that expanded and modified the earlier work of Gundry and Welsch (2001). Gundry and Welsch’s research was groundbreaking in its approach to defining and evaluating the attitudes that influence entrepreneurial behavior, with a particular emphasis on proactiveness, innovation, and risk-taking. Nabi and colleagues further elaborated on this by enhancing the scale to more accurately reflect the subtle attitudes that predict entrepreneurial intention and behaviors. The scale was modified to include a series of questions that were specifically designed to measure various aspects of entrepreneurial attitude, thereby guaranteeing its reliability and validity across a wide range of populations. The entrepreneurial attitude scale, which was developed by Nabi et al. (2011), was implemented in our investigation. This scale comprises five inquiries that are specifically designed to evaluate the attitudes of individuals toward entrepreneurship. Three sample queries from this scale are as follows: “I am enthusiastic about the prospect of becoming an entrepreneur,” “I am confident in my capacity to establish a business,” and “I am convinced that starting a business would be a beneficial experience.” These queries are intended to evaluate the confidence, positivity, and enthusiasm of respondents with respect to entrepreneurship, thereby offering valuable insights into their overall entrepreneurial attitude. Our research endeavors to enhance the current body of knowledge and offer a reliable assessment of entrepreneurial attitude by utilizing the validated and refined scale from prior studies.

A substantial factor loading was observed for each survey question that was intended to assess the entrepreneurial attitude construct, with values ranging from 0.723 to 0.841. The scale’s construct reliability of 0.813 was considerably higher than the evaluation threshold of 0.60, suggesting that it exhibited high internal consistency. Additionally, the scale’s AVE was 0.609, which exceeded the minimum requirement of 0.5, indicating that the scale has good discriminant validity and robust construct validity. The scale’s goodness of fit indices was also noteworthy, with GFI, AGFI, NFI, IFI, and CFI values of 0.911, 0.919, 0.967, and 0.934, respectively, and an RMSEA value of 0.031. Collectively, these findings suggest that the scale is a valid and dependable instrument for evaluating entrepreneurial attitudes and that it is well-suited to the data.

We ensured a robust and validated approach by adopting and adapting the queries from numerous influential previous studies to measure the construct of subjective norm. In particular, we drew inspiration from the research conducted by Xia et al. (2020), Nieuwenhuizen and Swanepoel (2015), and Liao and Fang (2019). Xia et al. (2020) offered a dependable set of questions that capture this dynamic, providing foundational insights into how perceived social pressures influence individuals’ intentions. Nieuwenhuizen and Swanepoel (2015) further refined these measures by contextualizing them within various cultural contexts and validating their relevance across diverse populations. The study conducted by Liao and Fang (2019) provided supplementary substance, particularly in the study of the subtleties of social influence in entrepreneurial contexts. We developed a comprehensive four-question scale that is specifically designed to accurately measure subjective norms by incorporating and adapting the questions from these studies. This scale evaluates the extent to which individuals believe that significant others, including family, acquaintances, and colleagues, anticipate their participation in entrepreneurial endeavors. The queries are designed to assess the degree to which these social influences influence the entrepreneurial intention of the respondents, thereby facilitating a more sophisticated comprehension of the social pressures and expectations they encounter. This modification guarantees that our measurement instrument is both valid and reliable, as it is based on well-established research and is customized to the unique context of our study. Three sample queries from this scale are as follows: “Do you believe that people who are important to you support your decision to become an entrepreneur?”, “Do you feel social pressure from those around you to pursue entrepreneurial activities?”, and “Do influential people in your life encourage you to pursue entrepreneurship as a career?”.

The factor loading of the survey questions measuring the construct of subjective norm ranged from 0.717 to 0.869, suggesting that there were significant correlations between the questions and the underlying construct. The scale’s internal consistency was confirmed by the construct reliability of 0.788, which exceeded the evaluation threshold of 0.60. Furthermore, the AVE was 0.603, which exceeded the minimum requirement of 0.5, indicating that the scale possesses exceptional discriminant validity and construct validity. The scale’s robustness was further substantiated by the goodness of fit test results, which showed a GFI of 0.913, AGFI of 0.909, NFI of 0.898, IFI of 0.914, and RMSEA of 0.039. Collectively, these values suggest that the scale is a valid and dependable instrument for assessing subjective norms and that it is well-suited to the data.

We employed a framework that was derived from the research conducted by Anggadwita and Dhewanto (2016) to assess PBC. Their research yielded a comprehensive collection of measures that were specifically designed to evaluate the perceptions of individuals regarding their capacity to engage in entrepreneurial activities. These measures encompassed critical elements such as self-efficacy, the availability of resources, and the perceived ease or difficulty of establishing and operating a business. The construct of PBC is of great importance in entrepreneurial intention models, as it is indicative of an individual’s confidence in their abilities and the degree to which they believe that external factors either facilitate or impede their entrepreneurial objectives. Our scale is comprised of four meticulously crafted inquiries that are derived from the validated questions of Anggadwita and Dhewanto (2016). These inquiries are intended to provide a thorough evaluation of this construct. The following are three sample questions from our adapted scale: “I am confident in my ability to start a business if I wanted to,” which measures self-efficacy related to entrepreneurship; “I have the resources and knowledge required to successfully start a business,” which assesses perceived resource availability; and “Starting a business would be easy and manageable for me,” which gauges the overall perceived ease of undertaking entrepreneurial activities. These inquiries are intended to capture the multifaceted nature of PBC, offering insights into the overall feasibility of engaging in entrepreneurial endeavors, as well as individuals’ confidence and perception of resource availability. Our study endeavors to guarantee the reliability and validity of the measures by utilizing the established framework from Anggadwita and Dhewanto (2016), thereby providing valuable data to the comprehension of entrepreneurial intention.

Significant factor loadings were observed in each survey question intended to assess the PBC construct, with values ranging from 0.731 to 0.791. The scale’s construct reliability was 0.708, which is significantly higher than the acceptable threshold of 0.60, suggesting that it has a high level of internal consistency. Furthermore, the AVE was 0.576, which exceeded the minimum requirement of 0.5, thereby confirming the scale’s strong discriminant and construct validity. The scale’s goodness of fit indices was also noteworthy, with a GFI of 0.969, AGFI of 0.912, NFI of 0.932, IFI of 0.912, and an RMSEA value of 0.049. Collectively, these findings suggest that the scale is a valid and dependable instrument for assessing PBC, as it closely aligns with the data and generates consistent measurements.

We created an index to assess family support that was derived from the exhaustive study conducted by Osorio et al. (2017). Their research offered a comprehensive framework for comprehending the various aspects of family support, particularly in the context of entrepreneurship. We designed five specific questions to capture the various aspects of family support, including emotional encouragement, financial assistance, and practical aid, in recognition of the significant influence that family support can have on an individual’s entrepreneurial journey. The index is intended to measure the extent and character of the support provided by family members to the individual’s entrepreneurial pursuits. For instance, emotional encouragement may be evaluated by examining the manner in which family members motivate and inspire the individual, while financial assistance queries may be used to determine the family members’ willingness to provide funding or financial resources. The availability of family members to provide expertise or assist with business-related duties would be the focus of practical help questions. Three sample questions from our index are as follows: “My family motivates me to pursue my business ideas,” which evaluates emotional support; “My family is prepared to offer financial support if necessary for my business,” which measures financial support; and “My family assists me with business-related tasks,” which evaluates practical support. The purpose of these inquiries is to obtain a comprehensive understanding of the family support that an individual receives, thereby offering valuable insights into the ways in which this support affects their entrepreneurial endeavors.

The survey questions that were intended to evaluate the family support construct exhibited factor loadings ranging from 0.709 to 0.813, suggesting that they were strongly correlated with the underlying construct. The scale’s construct reliability was 0.739, which is substantially higher than the acceptable threshold of 0.60, indicating a high level of internal consistency. Furthermore, the AVE was 0.727, superseding the minimum requirement of 0.5, thereby confirming the scale’s exceptional discriminant validity and construct validity. The scale’s goodness of fit indices was also noteworthy, with a GFI of 0.912, AGFI of 0.931, NFI of 0.978, IFI of 0.925, and an RMSEA value of 0.038. Collectively, these findings suggest that the scale is a valid and dependable instrument for assessing family support, with a satisfactory fit to the data.

In order to assess entrepreneurial self-efficacy, we implemented and customized the framework established by Tsai et al. (2016) and Linan (2008). These studies provided a comprehensive approach to quantifying and comprehending the confidence that individuals have in their entrepreneurial capabilities. Tsai and Chang’s research underscored the importance of self-efficacy in shaping entrepreneurial intention and behaviors, emphasizing critical components such as innovation, leadership, and problem-solving. These concepts were further refined by Linan’s research, which provided validated scales that have been extensively employed in entrepreneurial research. Our scale is composed of five meticulously crafted questions that are intended to capture a variety of aspects of entrepreneurial self-efficacy, all of which are based on these strong foundations. The purpose of these questions is to evaluate the confidence of individuals in their capacity to effectively complete entrepreneurship-related duties. For instance, an inquiry could be: “I am confident in my capacity to identify new business opportunities,” which assesses the individual’s faith in their ability to identify opportunities. An alternative inquiry could be: “I am capable of creating a business plan that will captivate investors,” which evaluates my confidence in resource mobilization and planning. A third inquiry could be: “I am capable of leading and managing a small business,” which assesses confidence in leadership and management. By incorporating these questions, our scale offers a comprehensive assessment of entrepreneurial self-efficacy, guaranteeing that it encompasses the critical components that were emphasized in the research conducted by Tsai et al. (2016) and Linan (2008). This method guarantees the measurement’s reliability and validity, while also offering valuable insights into the role of self-efficacy in shaping entrepreneurial intention and behaviors (Table 1).

**Table 1:** Latent variable measurement.

Latent variable	Quantity of inquiries	Theoretical support
Entrepreneurial intention	5 items	Liñán and Chen (2009) and Anggadwita and Dhewanto (2016)
Entrepreneurial attitude	4 items	Nabi et al. (2011)
Subjective norm	4 items	Xia et al. (2020), Nieuwenhuizen and Swanepoel (2015), and Liao and Fang (2019)
PBC	4 items	Anggadwita and Dhewanto (2016)
Family support	4 items	Osorio et al. (2017)
Entrepreneurial self-efficacy	5 items	Tsai et al. (2016) and Linan (2008)

### 4.2 Pilot study

A pilot study was undertaken by distributing 50 questionnaires to art and design students from several educational institutions in China’s Shandong, Jiangsu, and Zhejiang provinces. Out of 50 issued questionnaires, 44 valid replies were obtained, yielding an 88% response rate.

In our study, convenience sampling was used to collect information from art and design students at various educational institutions in the provinces of Shandong, Jiangsu, and Zhejiang. This sample strategy was chosen because it was practical and efficient in reaching a specific group of participants who were easily accessible and eager to participate in the study. Although convenience sampling has disadvantages, such as potential bias and lack of generalizability, it enabled us to swiftly gather preliminary data and insights into the factors impacting entrepreneurial inclinations among this distinct student group. The pilot study’s high response rate of 88% indicates that this strategy was successful in engaging students and gathering significant information for future research. Future research may employ more stringent sampling procedures to improve the representativeness and validity of the findings.

#### 4.2.1 Internal consistency

Cronbach’s alpha was utilized to measure the internal consistency of our study’s scales. The measures assessed a variety of factors, including family support, entrepreneurial self-efficacy, subjective norm, PBC, entrepreneurial attitude, and entrepreneurial purpose. The Cronbach’s alphas for these scales were 0.731 for family support, 0.712 for entrepreneurial self-efficacy, 0.724 for subjective norm, 0.736 for PBC, 0.802 for entrepreneurial attitude, and 0.867 for entrepreneurial intention. A Cronbach’s alpha score greater than 0.70 implies good internal consistency, implying that the items on each scale accurately measure the same underlying construct. This reliability indicates that respondents’ responses were consistent across multiple items within each test, rendering the data reliable and viable for future study. The high internal consistency across different scales contributes to the robustness of the measures, ensuring that the constructs are reliably captured and may be securely used to derive significant results in the study.

#### 4.2.2 Construct validity

To establish construct validity in this study, both exploratory factor analysis (EFA) and confirmatory factor analysis (CFA) were used. The EFA was initially performed to investigate the underlying structure of the scales and validate the proposed factor structure of the original scale. This stage entailed determining the number of factors and the elements that load onto each component, so giving an empirical foundation for the scales employed. Following the EFA, CFA was used to confirm the factor discovered during the EFA, confirming that the scales appropriately measured the desired structures. The Kaiser-Meyer-Olkin (KMO) index and Bartlett’s sphericity test both confirmed the factor structure’s validity. The KMO index returned to a value of 0.933, suggesting that the data were appropriate for factor analysis, as values more than 0.90 are deemed excellent. Furthermore, Bartlett’s test of sphericity yielded a significant result (χ^2^ = 313.45, *p*-value<0.001), showing sufficient correlations between items for EFA. These statistical tests demonstrate that the dataset was suitable for factor analysis, and that the scales utilized in this study have strong construct validity. This rigorous validation procedure guarantees that the constructs of family support, entrepreneurial self-efficacy, subjective norm, PBC, entrepreneurial attitude, and entrepreneurial intention are measured precisely and consistently.

### 4.3 Main data collection

Determining an appropriate sample size is essential to ensure adequate statistical power and the ability to detect meaningful effects in empirical research. Numerous techniques exist for estimating optimal sample sizes, with G*Power widely regarded as a robust and contemporary tool for conducting power analyses in social science and behavioral research (Faul et al., 2007). Scholars have proposed several conventions for selecting effect sizes; for instance, Selya et al. (2012) classify effect sizes of 0.10, 0.15, and 0.35 as small, medium, and large effects, respectively.

**Figure 2:**
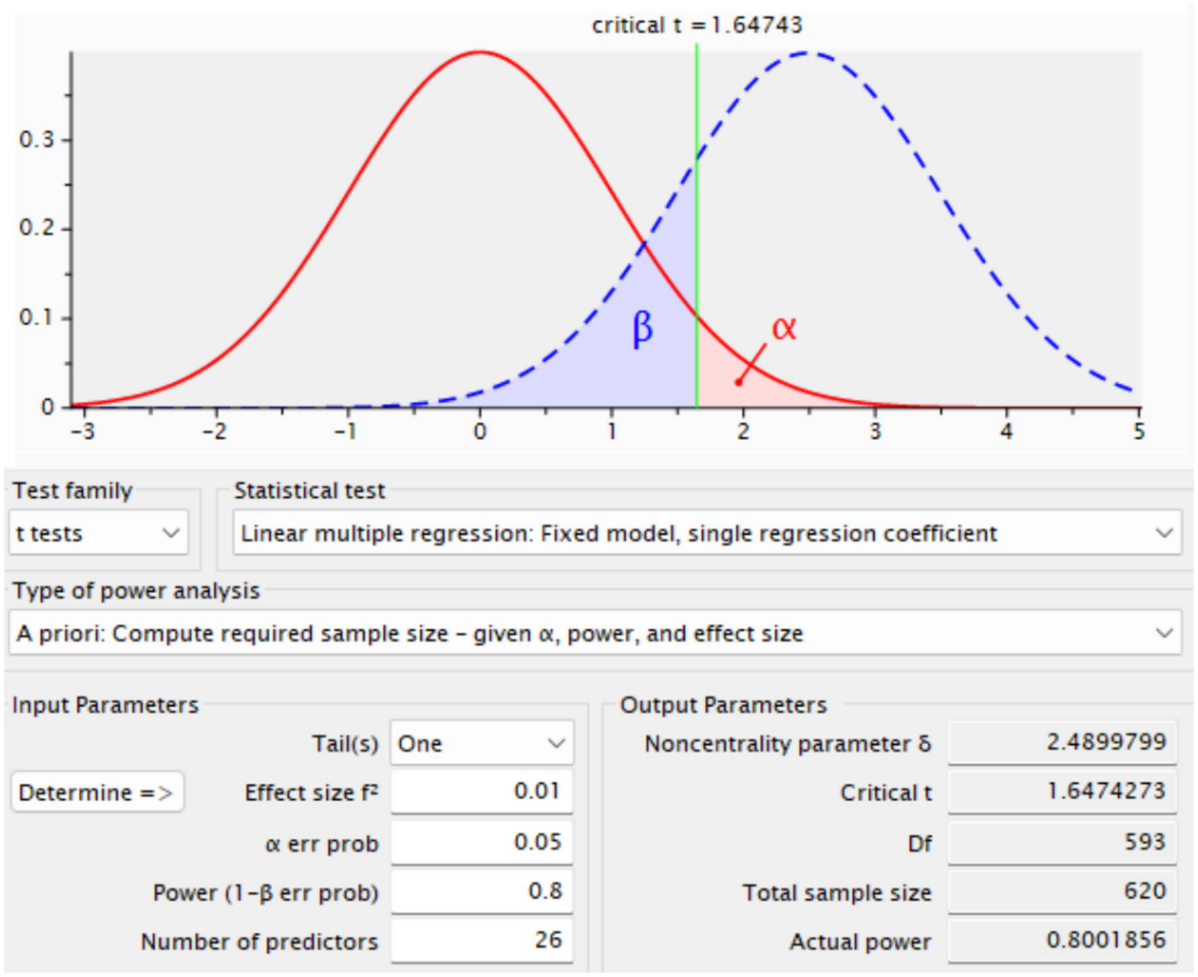
G*Power outputs.

In this study, however, a conservative approach was adopted by choosing a smaller effect size of 0.05. Using a smaller effect size increases the required sample size but strengthens the analysis by reducing the risk of Type II errors and enhancing the generalizability and robustness of the findings (Maher et al., 2013). Based on 26 predictors, an alpha level of 0.01, and 80% statistical power—parameters consistent with rigorous standards in social science research—the minimum required sample size for the present study was calculated to be 620 participants (Figure 2).

The survey instrument was initially developed in English and subsequently evaluated by two bilingual experts fluent in both Chinese and English to ensure linguistic accuracy and cultural relevance. To further ensure translation quality, a standard translation and back-translation procedure was employed, allowing for verification of semantic equivalence and clarity in the finalized Chinese version of the questionnaire.

The study was conducted across 25 universities in China offering undergraduate and postgraduate programs in art and design. Prior to data collection, each institution was formally contacted through established administrative channels, with communication directed to department heads and relevant research administrators. Detailed information on the study’s aims, scope, and ethical approval was provided, and all research activities were conducted in accordance with national regulations and institutional ethical guidelines.

Due to constraints related to institutional access and the widespread use of WeChat as a primary academic communication platform in Chinese higher education, a non-probability convenience sampling strategy was adopted. While WeChat enables efficient survey dissemination and access to large student populations, its use also presents methodological limitations. Participation depends on students’ membership in specific academic or social groups, which may affect sample representativeness. Students with lower engagement with social media or limited access to institutional WeChat channels may be underrepresented, and distributing surveys in groups may introduce subtle social influence effects. In addition, reliance on online platforms may exclude individuals with limited internet access or who prefer not to participate digitally. These factors should be considered when interpreting the generalizability of the findings.

To enhance sample coverage and diversity, the questionnaire was administered through the Wen Juan Xing platform, with survey links distributed via university-affiliated WeChat groups, departmental forums, and class communication channels coordinated by lecturers and student representatives. This recruitment approach aligns with established practices in large-scale research within Chinese higher education, where digital platforms commonly facilitate academic coordination and research participation. Participation was voluntary, and no incentives were provided to minimize potential response bias. The resulting sample exhibited substantial variation in academic level, discipline, gender, and institutional affiliation, supporting its suitability for subsequent analyses.

Data was collected over three consecutive academic semesters to capture longitudinal patterns in the study variables. Participants included undergraduate and postgraduate art and design students who were expected to remain enrolled for at least three additional semesters at the time of the initial survey. The first wave of data collection (T1) occurred between August and September 2024, followed by the second wave (T2) between February and March 2025, and the third wave (T3) between August and September 2025. Undergraduate participants were required to be in at least their third semester at T1 to ensure sufficient academic exposure, while postgraduate students in their first or second semester were eligible, provided their expected enrollment extended across all waves. A total of 853 participants completed the survey at T1, 826 remained at T2, and 786 completed all three waves. To ensure consistency and completeness of longitudinal data, the final analytical sample consisted of 786 participants who provided valid responses at all time points.

## 5 Results

### 5.1 Descriptive statistics

Based on the 786 valid responses, 44.2% of the participants were male (*n* = 347) and 55.8% were female (*n* = 439). With respect to educational level within the art and design sample, 39.26% of the participants were enrolled in undergraduate programs (*n* = 308), 33.13% were master’s students (*n* = 260), 24.9% were enrolled in other programs (*n* = 196), and 1.61% were doctoral students (*n* = 13).

In terms of age distribution, participants aged 18–25 years accounted for 42.5% of the sample (*n* = 334), those aged 26–35 years comprised 42.19% (*n* = 332), participants aged 36–45 years represented 16.36% (*n* = 129), and individuals aged 45 years and above constituted 1.21% of the sample (*n* = 10). Regarding family entrepreneurial background, 77% of the participants (*n* = 605) reported having a family history of entrepreneurship, whereas 23% (*n* = 181) reported no such background.

### 5.2 Reliability and validity

Fornell and Larcker (1981) determined the validity and reliability of SEM analysis based on the following terms and conditions:

(a) Reliability:

- For every latent variable, the factor loading of the measurement variable should be higher than 0.70.
- For every latent variable, the average variance extracted should be equal to or higher than 0.50.

(b) Validity:

For every latent variable, the Cronbach’s alpha value should be equal to or higher than 0.7.

Table 2 presents the validity and reliability analyses based on Cronbach’s alpha, factor loading, and average variance extracted research variables. As for all the latent variables, the average variance extracted was greater than 0.5 and the Cronbach’s alpha was higher than 0.7, and factor loadings were higher than 0.7. Therefore, the reliability and validity of the research model were accepted.

**Table 2:** Analyses of research validity and reliability.

Parameter description	Factor loading	Cronbach’s alpha	AVE
Entrepreneurial Intention-T3	[0.712, 0.801]	0.781	0.583
Subject Norm-T2	[0.725, 0.818]	0.842	0.628
Entrepreneurial Attitude-T2	[0.731, 0.846]	0.724	0.701
PBC-T2	[0.718, 0.789]	0.856	0.566
Entrepreneurial Self-Efficacy-T2	[0.728, 0.862]	0.826	0.689
Family Support-T1	[0.717, 0.809]	0.885	0.701

### 5.3 Structural model

Through the BSEM method, the structural model aids the process of recognizing the hypothesized relationships between the variables that show links to the presumed model’s conception. Moreover, Table 3 presents the output of SEM.

**Table 3:** Hypotheses testing.

Path	Beta	*p*-value	Result
Direct effects			
H_1_: Entrepreneurial attitude (T2) → Entrepreneurial intention (T3)	0.41	< 0.001	Significant
H_2_: Subjective norm (T2) → Entrepreneurial intention (T3)	0.08	0.284	Non-Significant
H_3_: PBC (T2) → Entrepreneurial intention (T3)	0.31	0.019	Significant
H_4_: Family Support (T1) → Entrepreneurial intention (T3)	0.48	< 0.001	Significant
H_5_: Family Support (T1) → Entrepreneurial Self-efficacy (T2)	0.66	< 0.001	Significant
H_6_: Family Support (T1) → PBC (T2)	0.59	< 0.001	Significant
H_7_: Entrepreneurial Self-efficacy (T2) → PBC (T2)	0.36	< 0.001	Significant
H_8_: Entrepreneurial Self-efficacy (T2) → Entrepreneurial intention (T3)	0.62	< 0.001	Significant
Indirect effect			
H_9_: Family Support (T1) → Entrepreneurial Self-efficacy (T2) → Entrepreneurial intention (T3)	0.409	<0.001	Significant

The outputs from the SEM analysis reveal several significant relationships among the factors influencing entrepreneurial intention among art and design students. Firstly, the hypothesis that entrepreneurial attitude positively affects entrepreneurial intention (H1) is supported with a significant coefficient of 0.41 (*p* < 0.001), indicating that students with positive attitudes toward entrepreneurship are more likely to develop strong entrepreneurial intention. Conversely, the hypothesis regarding subjective norm’s impact on entrepreneurial intention (H2) is not supported, as the relationship is weak (coefficient of 0.08) and non-significant (*p* = 0.284), suggesting that perceived social pressure from family and peers does not significantly influence these students’ entrepreneurial intention. The hypothesis that PBC influences entrepreneurial intention (H3) is confirmed with a significant coefficient of 0.31 (*p* = 0.019), highlighting the importance of self-confidence in entrepreneurial capabilities. Furthermore, family support is found to significantly impact entrepreneurial intention (H4) with a coefficient of 0.48 (*p* < 0.001), entrepreneurial self-efficacy (H5) with a coefficient of 0.66 (*p* < 0.001), and PBC (H6) with a coefficient of 0.59 (*p* < 0.001). Additionally, entrepreneurial self-efficacy significantly affects PBC (H7) with a coefficient of 0.36 (*p* < 0.001) and entrepreneurial intention (H8) with a coefficient of 0.62 (*p* < 0.001). Finally, the mediation analysis confirms that entrepreneurial self-efficacy partially mediates the relationship between family support and entrepreneurial intention, with a significant indirect effect (coefficient of 0.409, *p* < 0.001), demonstrating the critical role of self-efficacy in translating family support into entrepreneurial intention.

## 6 Discussion

This study examined the associations between family support and entrepreneurial intention among college students, providing focused evidence on how these factors are related within an art and design student population. Building on prior research, the study examined the relationships among family support, entrepreneurial self-efficacy, and entrepreneurial intention, highlighting the role of family support in this specific educational context. By integrating SCT with the extended TPB, the study offers a more nuanced understanding of how social and psychological factors jointly relate to entrepreneurial intention in creative disciplines. In addition, the study assessed the mediating role of entrepreneurial self-efficacy in the association between family support and entrepreneurial intention, offering further insight into the psychological processes linking social support to entrepreneurial motivation.

The research framework comprised six latent variables and 26 observed indicators, organized around nine research hypotheses. By combining SCT with the extended TPB, the study aimed to provide a comprehensive examination of factors associated with entrepreneurial intention. The proposed model was evaluated using SEM, which enabled an examination of relationships among variables and the assessment of indirect associations within the model. The findings indicated that the integrated SCT and extended TPB framework is useful for understanding patterns of entrepreneurial intention, underscoring the relevance of family support and entrepreneurial self-efficacy within the art and design education context. This integrative approach offers a structured framework for examining entrepreneurial intention in this specific educational setting.

The discussion is organized into three sections corresponding to hypotheses derived from the TPB, the extended TPB, and the integrated extended TPB with SCT.

### 6.1 Discussion of hypotheses H1, H2, H3 (TPB)

The first three hypotheses (H1, H2, and H3) represent the core hypotheses commonly examined in entrepreneurial intention research grounded in the TPB framework. The first hypothesis (H1) examines the association between attitude and entrepreneurial intention, proposing that more favorable attitudes toward entrepreneurship are associated with stronger entrepreneurial intention. The results indicate a significant association, consistent with prior studies by Lyu et al. (2024), Tran et al. (2024), and Chen (2024), who reported that positive entrepreneurial attitudes are associated with entrepreneurial intention. These findings suggest that individuals holding favorable views toward entrepreneurship tend to report higher levels of entrepreneurial intention, reflecting stronger interest and positive perceptions of entrepreneurial activity. This result highlights the relevance of attitude in relation to entrepreneurial intention and underscores the importance of fostering favorable entrepreneurial attitudes among prospective entrepreneurs. Among art and design students, perceiving entrepreneurship as a viable and appealing career option may be associated with higher levels of confidence and engagement with entrepreneurial pursuits. Prior research also suggests that exposure to entrepreneurial concepts and success stories within educational settings is associated with more proactive entrepreneurial orientations and higher reported motivation to pursue entrepreneurial activities (Perez et al., 2024).

With respect to subjective norm, defined as perceived social pressure to engage or not engage in a particular behavior, the findings indicate no significant association with entrepreneurial intention (H2) among art and design students. This result is consistent with previous studies by Anderson (2023), Pham et al. (2023), and Maydiantoro et al. (2021), which similarly reported non-significant associations between subjective norms and entrepreneurial intention. These findings point to the contextual variability of factors related to entrepreneurial intention and suggest that the relevance of subjective norms may differ across populations and disciplinary contexts. Art and design students often prioritize personal vision, creative autonomy, and self-expression, which may reduce the salience of external social expectations in shaping entrepreneurial intention. In addition, individuals in creative disciplines may receive mixed or ambiguous social signals regarding the desirability and feasibility of entrepreneurship, which may further weaken the association between subjective norm and entrepreneurial intention.

The third hypothesis (H3) examined the association between PBC and entrepreneurial intention. The findings indicate a significant positive association, in line with prior research (Lyu et al., 2024; Tran et al., 2024), suggesting that individuals who perceive higher levels of control over entrepreneurial behaviors tend to report stronger entrepreneurial intention. PBC reflects individuals’ perceptions of feasibility and control regarding entrepreneurial activities, including their confidence in managing tasks and challenges associated with business initiation and operation.

The non-significant association between subjective norm and entrepreneurial intention warrants further discussion. One potential explanation relates to the nature of creativity-oriented career pathways pursued by art and design students. Compared with business or engineering careers, creative careers are often less standardized and more strongly oriented toward individuality and personal identity. Consequently, entrepreneurial decisions in creative fields may be more closely aligned with internal motivations and self-concept than with perceived social expectations, thereby reducing the relative importance of normative social pressure.

In addition to disciplinary culture, methodological and contextual considerations may also contribute to the non-significant role of subjective norm. Within the present model, family support represents a salient and proximal form of social influence that provides emotional and instrumental resources. This construct may overlap conceptually with broader normative perceptions captured by subjective norm, thereby attenuating the unique association between subjective norm and entrepreneurial intention when both variables are included simultaneously. Although multicollinearity diagnostics indicated acceptable variance inflation factors, conceptual proximity between family-related social influence and subjective norm may still reduce the distinct explanatory contribution of subjective norm. Moreover, subjective norms may be indirectly related to entrepreneurial intention through psychological factors such as entrepreneurial self-efficacy, rather than being directly associated with intention, particularly in creative disciplines where social approval is less consistent and more context dependent.

### 6.2 Discussion of hypotheses H4, H5, H6 (extended TPB)

Family support is an important element in the extended TPB, which incorporates broader social influences into the model. This study proposed three hypotheses to examine the role of family support: H4 examines the association between family support and entrepreneurial intention, H6 examines the association between family support and PBC, and H7 examines the association between family support and entrepreneurial self-efficacy.

The results indicate a positive association between family support and entrepreneurial intention (H4), which is consistent with previous studies by Annisa et al. (2021) and Halizah and Mardikaningsih (2022). Prior research suggests that family support is closely related to entrepreneurial intention. One possible interpretation is that emotional support and positive reinforcement from family members are associated with higher levels of self-confidence and self-esteem. When individuals perceive emotional support from their families, they tend to report stronger beliefs in their own abilities (Molino et al., 2018). Such perceived confidence is often associated with stronger entrepreneurial intention. In addition, financial assistance or access to resources provided by family members may be associated with lower perceived financial barriers and anxiety related to business initiation. This perceived feasibility may be linked to higher levels of entrepreneurial intention.

The findings further indicate that family support is positively associated with entrepreneurial self-efficacy (H5) and that entrepreneurial self-efficacy is positively associated with entrepreneurial intention (H6), in line with earlier research (Pham et al., 2023; Saoula et al., 2023; Suhartanto, 2022; Wardana et al., 2024). These results are consistent with Li et al. (2023), who reported that family support is associated with higher levels of confidence in entrepreneurial capability. Family support may involve emotional encouragement as well as access to practical resources and mentorship, which are associated with stronger self-efficacy beliefs. When family members express encouragement and confidence in an individual’s entrepreneurial goals, this perception is often related to higher motivation to pursue entrepreneurship. Perceived family validation may reinforce individuals’ beliefs that they possess the skills and abilities necessary for entrepreneurial activity.

Family support has also been discussed in relation to PBC. Material and emotional resources provided by family members are frequently associated with stronger perceptions of control over entrepreneurial activities (Halizah and Mardikaningsih, 2022). Financial assistance, advice, and task-related support may be linked to higher perceived access to essential entrepreneurial resources. Emotional support may be associated with lower perceived stress and anxiety, which in turn corresponds with stronger perceptions of control over entrepreneurial actions.

### 6.3 Discussion of hypotheses H7, H8, H9 (integrated extended TPB with SCT)

Self-efficacy, one of the primary psychological constructs examined in this study, plays a central role in integrating the extended TPB and SCT within the theoretical framework. Hypotheses 7 and 8 examined the associations among entrepreneurial self-efficacy, PBC, and entrepreneurial intention. SEM results indicated that entrepreneurial self-efficacy is significantly associated with both PBC and entrepreneurial intention, consistent with earlier studies by Wardana et al. (2024), Christensen et al. (2023), and Taneja et al. (2024). In addition, the findings suggest that entrepreneurial self-efficacy partially mediates the association between family support and entrepreneurial intention (H9), extending prior research by highlighting an indirect pathway linking social support to entrepreneurial intention.

Several interpretations may account for these observed associations among art and design students. First, self-efficacy in this population is often closely linked to creative confidence and perceived capacity to generate and implement novel ideas. Such beliefs are commonly associated with stronger perceptions of control over entrepreneurial tasks (PBC), as students perceive themselves as more capable of managing uncertainty and challenges. Second, the collaborative and supportive learning environments typical of art and design education are frequently associated with higher self-efficacy. Peer feedback, mentorship, and communities that value creativity provide ongoing reinforcement that may be related to stronger entrepreneurial intention. Third, project-based learning and real-world application of skills are commonly embedded in art and design curricula and are associated with higher self-efficacy and entrepreneurial orientation. Together, these patterns suggest that entrepreneurial self-efficacy is related to both entrepreneurial intention and perceived control, and that it serves as an intervening psychological construct in the association between family support and entrepreneurial intention.

To further clarify the psychological transmission underlying the mediating role of entrepreneurial self-efficacy, it is useful to consider how family support is linked to efficacy beliefs. Drawing on SCT, family support may be associated with entrepreneurial self-efficacy through multiple pathways, including verbal encouragement, emotional reassurance, and early mastery-related experiences. Perceived encouragement and validation from family members are related to stronger beliefs in entrepreneurial capability, while emotional support is associated with lower perceived fear of failure and uncertainty. In addition, instrumental assistance or role modeling provided by family members may be linked to early success experiences, which correspond with stronger efficacy beliefs. These mechanisms suggest that family support functions as a psychological resource rather than solely as a contextual factor.

Although entrepreneurial self-efficacy and PBC are related constructs, they represent conceptually distinct psychological processes. Entrepreneurial self-efficacy reflects individuals’ beliefs about their internal capability to perform entrepreneurial tasks, whereas PBC captures perceptions of controllability and feasibility in relation to external constraints such as resources, opportunities, and structural barriers. In this sense, self-efficacy can be understood as a belief that is conceptually related to, but not redundant with, perceived control. This distinction is particularly relevant in creative disciplines, where confidence in creative and entrepreneurial abilities may precede or coexist with uncertainty regarding environmental feasibility. Clarifying this distinction strengthens the conceptual logic of the mediation model and supports the theoretical integration of SCT and the extended TPB.

The psychological patterns identified in this study may differ from those observed among business or engineering students, given the distinctive cognitive orientations and learning environments of art and design disciplines. Unlike business and engineering students, who are often trained in analytical reasoning and technical problem-solving, art and design students operate within creativity-oriented contexts emphasizing originality, self-expression, and aesthetic judgment. Consequently, entrepreneurial self-efficacy among art and design students is more closely associated with creative confidence and perceived autonomy than with technical competence or market efficiency. Family support may therefore be particularly salient by legitimizing creative career pathways that are often perceived as uncertain or unconventional, thereby relating to stronger confidence in translating creative skills into entrepreneurial pursuits. This disciplinary context may help explain why entrepreneurial self-efficacy functions as a more prominent psychological bridge between family support and entrepreneurial intention in art and design education than is typically reported in business or engineering contexts, where institutional norms and professional training may serve similar roles.

Although entrepreneurial self-efficacy and PBC are conceptually related, their simultaneous inclusion in the model is theoretically justified. Entrepreneurial self-efficacy reflects beliefs about personal capability, whereas PBC captures perceptions of feasibility and controllability considering external constraints. Distinguishing these constructs allows the model to differentiate internal confidence from contextual feasibility, which are emphasized differently within SCT and TPB. In art and design education, this distinction is particularly meaningful, as students may report strong confidence in their creative abilities while simultaneously expressing uncertainty regarding external conditions. Accordingly, establishing discriminant validity between entrepreneurial self-efficacy and PBC is not only statistically appropriate but also theoretically important for clarifying their distinct psychological roles in relation to entrepreneurial intention.

### 6.4 Theoretical implications

Understanding the factors associated with entrepreneurial inclination is important for informing educational programs and policy discussions related to entrepreneurship. This study integrates the extended TPB and SCT to provide a comprehensive framework for examining these factors. By incorporating entrepreneurial self-efficacy and family support, the study offers a more nuanced understanding of the psychological and social factors related to entrepreneurial intention, particularly among art and design students. The findings yield several theoretical implications.

1. *Integration of extended TPB and SCT*: This study combines the extended TPB with SCT to provide a more comprehensive conceptual framework for understanding entrepreneurial intention. Integrating these two theories facilitates a clearer examination of how psychological and social factors are jointly related to entrepreneurial intention. This approach enables more detailed consideration of how attitudes, subjective norms, PBC, and self-efficacy coexist within a unified framework, offering a useful reference point for future research.
2. *Role of entrepreneurial self-efficacy*: The findings highlight the relevance of entrepreneurial self-efficacy in relation to both PBC and entrepreneurial intention. This underscores self-efficacy as an important psychological construct that merits inclusion in models of entrepreneurial intention. The study contributes to the literature by showing that entrepreneurial self-efficacy is directly associated with entrepreneurial intention and serves as an intervening variable in the relationship between family support and entrepreneurial intention. This mediating role suggests that self-efficacy is a meaningful construct for consideration in entrepreneurship education and research.
3. *Family support as an extension of TPB*: The inclusion of family support within the extended TPB framework emphasizes its relevance as a social contextual factor associated with entrepreneurial intention. The results indicate that family support is directly related to entrepreneurial intention and indirectly related through its association with entrepreneurial self-efficacy. This extension of the TPB highlights the importance of social and familial contexts in entrepreneurship research and suggests that these influences warrant greater consideration in future theoretical and empirical studies.
4. *Contextual focus on art and design students*: By focusing on art and design students, the study provides context-specific insights into how entrepreneurial intention is expressed within creative disciplines. The prominent role of entrepreneurial self-efficacy in this setting suggests that confidence in creative and entrepreneurial capabilities is closely related to entrepreneurial intention among these students. This contextual focus indicates that the integrated TPB–SCT framework may be applicable across disciplines, while also allowing for discipline-specific interpretations.
5. *Mediating role of entrepreneurial self-efficacy*: Identifying entrepreneurial self-efficacy as a partial mediator in the association between family support and entrepreneurial intention contributes to the existing literature. These findings advance understanding of how social support is psychologically internalized in relation to entrepreneurial intention. It suggests that self-efficacy represents a key psychological pathway through which social contexts are associated with entrepreneurial motivation.
6. *Cultural context and generalizability*: Although this study is situated within the Chinese cultural context, some findings may be relevant beyond national boundaries, while others should be interpreted with cultural sensitivity. The strong association between family support and entrepreneurial intention reflects the collectivist orientation of Chinese society, where family often plays a central role in career decision-making, risk sharing, and emotional support. In more individualistic or Western contexts, the role of family support may be less pronounced or replaced by alternative forms of social support, such as peer networks, mentors, or institutional resources. Nevertheless, the core psychological constructs examined in this study, particularly entrepreneurial self-efficacy and PBC, are theoretically grounded in SCT and TPB and have received empirical support across diverse cultural settings. This suggests that while sources of support may vary across cultures, the underlying motivational processes linking self-belief and perceived control to entrepreneurial intention may be cross-cultural relevant. Future cross-cultural research could further explore how different social agents complement or substitute for family support in varying institutional and cultural contexts.

### 6.5 Practical implications

The study’s findings offer several practical implications for educators, policymakers, and practitioners seeking to support entrepreneurial intention among art and design students.

(1) *Entrepreneurship education design*: Incorporating entrepreneurial self-efficacy into curricula may be associated with higher levels of student confidence and motivation toward entrepreneurial engagement. Educational programs may benefit from emphasizing self-efficacy development through experiential learning approaches, such as project-based activities, workshops, and mentorship opportunities, which allow students to apply creative skills in entrepreneurial contexts. Such approaches are commonly discussed as relevant for supporting entrepreneurial readiness and mindset development (Saoula et al., 2023).
(2) *Family involvement in entrepreneurship education*: Given the observed relevance of family support, institutions may consider involving family members in entrepreneurship-related initiatives. Workshops or informational sessions for families could highlight the role of encouragement and support in students’ entrepreneurial pathways and provide guidance on how families may offer constructive assistance. This approach may contribute to a more supportive social environment for students’ entrepreneurial intentions (Discua Cruz et al., 2013).
(3) *Targeted support for creative disciplines*: Art and design students often face discipline-specific challenges that can be addressed through tailored support mechanisms, such as incubators or accelerators focused on creative industries. These services may offer access to industry networks, funding information, and business development resources that are particularly relevant to creative entrepreneurial contexts (Zeng et al., 2023).
(4) *Policy considerations and support structures*: Policymakers may consider developing policy frameworks that support entrepreneurship in creative sectors. Measures such as grants, subsidies, or tax incentives for arts- and design-related ventures are frequently discussed as relevant forms of institutional support. In addition, regulatory environments that reduce procedural complexity may be associated with more favorable conditions for entrepreneurial participation among students in creative fields (Bazan et al., 2020).
(5) *Mentorship and role models*: Mentorship programs that connect students with experienced entrepreneurs in creative industries can provide valuable professional support. Exposure to role models may help students gain insights into entrepreneurial pathways, navigate industry challenges, and develop more informed expectations about entrepreneurial careers (Kong et al., 2020).

### 6.6 Limitations and future research

#### 6.6.1 Limitations

- While the sample size is large, there is a significant gender gap among the participants. This imbalance may have an impact on the findings, as entrepreneurial intention and self-efficacy differ significantly between genders.
- One of the study’s primary drawbacks is the sample’s exclusive emphasis on art and design students in a few Chinese provinces. While this provides useful insights into this specific demography, the conclusions are limited in their applicability to other student demographics or geographic regions.
- The use of convenience sampling limits the external validity of the findings. Although the sample includes art and design students from multiple institutions, participants were not randomly selected, and the results may not be representative of all art and design students in China or in other cultural contexts. Consequently, the findings should be interpreted as applicable primarily to populations with similar educational, cultural, and institutional characteristics.
- Using self-reported data can add bias because participants may give socially desired responses or may not precisely recollect their experiences and intentions.
- Although the study includes extended TPB and SCT, the measuring of factors such as family support, self-efficacy, and entrepreneurial purpose might be improved.
- One of the study’s weaknesses is the sample size, which is spread throughout three provinces: Shandong, Jiangsu, and Zhejiang. While the current sample size provides a significant quantity of data, increasing the number of participants would strengthen the findings and allow for more extensive comparison research across provinces. The current sample may not capture all regional variations in entrepreneurial intention and influencing factors, resulting in an imperfect knowledge of province disparities.
- Although the longitudinal design strengthens temporal ordering, the possibility of endogeneity cannot be fully excluded. Students with stronger entrepreneurial intention may be more likely to perceive or interpret family reactions as supportive, leading to potential reverse causality. Thus, perceived family support may partially reflect students’ existing motivational orientations rather than functioning solely as an antecedent of entrepreneurial intention.

#### 6.6.2 Future research

- Future research should use a more gender-balanced sample to guarantee that the results are representative and to investigate potential gender variations in entrepreneurial intention.
- Future study should broaden the demographic and geographic reach to include students from a variety of disciplines and places. Comparative research across cultural contexts can provide more information about how cultural and contextual factors influence entrepreneurial intention and practices.
- Future research employing probability-based sampling strategies and longitudinal designs (with different waves) would be valuable for strengthening generalizability and for examining how associations among family support, entrepreneurial self-efficacy, and entrepreneurial intention evolve over time. Longitudinal approaches may help identify critical periods when changes in self-efficacy and family support are most salient in the development of entrepreneurial intention across populations.
- Future research should focus on developing and evaluating interventions to boost entrepreneurial self-efficacy and family support. Experimental designs that execute and evaluate the efficacy of these treatments can offer educators and policymakers useful suggestions.
- Including variables like personality traits, emotional intelligence, and digital literacy could help us better understand the elements that influence entrepreneurial inclinations. Research into how these characteristics interact with self-efficacy and family support would provide a more complete view of the entrepreneurial process.
- Given the rapid growth of technology, future study should look into how digital tools and online platforms influence entrepreneurial intents and activities. Understanding how technology facilitates or hinders entrepreneurship among art and design students could help to shape relevant educational programs and legislation.
- Future research should focus on increasing the sample size to provide a more comprehensive comparison of the three provinces. A bigger and more evenly distributed sample from Shandong, Jiangsu, and Zhejiang provinces would allow researchers to conduct more robust statistical analyses and find regional variances and similarities in entrepreneurial inclinations. A comparison study of this nature could demonstrate how province economic situations, cultural characteristics, educational systems, and local support structures all have varied effects on entrepreneurial inclinations. This technique could also assist policymakers and educators in developing specialized entrepreneurship support initiatives based on each province’s specific requirements and characteristics.
- Future research could apply cross-lagged panel models, instrumental variable approaches, or experimental designs to more rigorously address potential endogeneity between perceived family support and entrepreneurial intention.

## Data Availability

The raw data supporting the conclusions of this article will be made available by the authors, without undue reservation.
